# Tuberculosis Microepidemics among Dispersed Migrants, Birmingham, UK, 2004–2013

**DOI:** 10.3201/eid2103.140209

**Published:** 2015-03

**Authors:** Melinda L. Munang, Catherine Browne, Shaina Khanom, Jason T. Evans, E. Grace Smith, Peter M. Hawkey, Heinke Kunst, Steven B. Welch, Martin J. Dedicoat

**Affiliations:** Heart of England NHS Foundation Trust, Birmingham, UK (M.L. Munang, C. Browne, S.B. Welch, M.J. Dedicoat);; Public Health England Regional Centre for Mycobacteriology, Birmingham (S. Khanom, J.T. Evans, E.G. Smith, P.M. Hawkey);; Queen Mary University of London, London, UK (H. Kunst)

**Keywords:** tuberculosis, molecular epidemiology, contact tracing, human migration, United Kingdom, UK, migrants, microepidemics, tuberculosis and other mycobacteria, bacteria

## Abstract

To determine if local transmission was responsible for rising tuberculosis incidence in a recently dispersed migrant community in Birmingham, UK, during 2004–2013, we conducted enhanced epidemiologic investigation of molecular clusters. This technique identified exact locations of social mixing and chains of apparent recent transmission, which can be helpful for directing resources.

For tuberculosis (TB) control programs, detection of recently acquired infection provides opportunities to stop ongoing transmission. As a proxy for recent infection, shared genotyping patterns are widely used (*1*–*3*). Universal genotyping of mycobacterial strains has been made possible by high-throughput, 24-loci mycobacterial repetitive-unit variable-number tandem-repeat (MIRU-VNTR) typing (*4*). However, inferring genuine recent transmission is complex. False molecular clusters can arise from reactivation of latent infection acquired in the distant past, limited strain variation over long periods, or lengthy stability of MIRU-VNTR markers (*5*). Among migrants from TB-endemic countries, determining whether TB infection was acquired locally or in the country of origin is difficult. We investigated TB incidence in a recently dispersed migrant community in Birmingham, UK, by using conventional and molecular epidemiologic techniques to define possible chains of recent transmission.

Birmingham has ≈1.1 million residents, of whom 20% were born abroad (*6*). In 2012, the local TB incidence was 58 cases per 100,000 population; 70% of cases were in foreign-born persons (Birmingham and Solihull TB Service, unpub. data). Birmingham has an established migrant community from the Indian subcontinent. In the past decade, a wave of new communities settled in Birmingham after implementation of a compulsory dispersal policy for asylum seekers (*7*), which aimed to ease housing and other social pressures in London and southeastern England (*8*). Changing migration patterns may have altered TB patterns; since 2005, incidence rates have stabilized in London but have been increasing in urban centers outside London (*9*). 

## The Study

In February 2012, the Birmingham and Solihull TB Service noted 2 distinct 24-loci MIRU-VNTR strain types of *Mycobacterium tuberculosis* (clusters A and B) isolated from 4 Eritrea-born patients with pulmonary TB. The diagnoses were made in July (cluster A) and December 2011 (cluster B). No epidemiologic links between any clustered patients were detected by routine contact investigation. Epidemiologic links were considered to be patients naming each other as contacts, sharing a contact without naming each other, or sharing a location during the infectious period of 1 patient. Case managers interviewed patients to elicit names of persons they may have had regular contact with over the infectious period; contacts in nonhousehold settings (e.g., workplace, leisure sites) were also included, but geographic locations were not always defined. The infectious period was defined as the date of onset of respiratory symptoms, if known, or 3 months before diagnosis.

Surveillance data revealed an increased number of Eritrea-born TB patients (1, 4, and 19 cases in 2004, 2006, and 2011 respectively); estimated incidence was 960 cases per 100,000 persons. To investigate whether transmission had occurred in the United Kingdom and to identify opportunities for wider community intervention, we invited all Eritrea-born TB patients (with and without strain typing available) reported during 2004–August 2012 for an extended interview with a dedicated TB nurse. Semistructured questions examined potential sources of infection and secondary cases. To systematically explore locations frequented by patients, nurses asked patients to complete a 24-hour work/rest/play diary. Since November 2011, extended interviews have been routinely conducted for all MIRU-VNTR clustered TB patients in Birmingham; no ethics approval was required. According to routine practice, clustered Eritrea-born patients continued to be interviewed through December 2013. All participants gave written consent.

During 2004–2013, a total of 88 cases occurred among Eritrea-born persons. There were no UK-born Eritrean (born to Eritrean parents) patients. Most patients were male (65%) and had pulmonary TB (67%); median age was 29 (interquartile range 25–35) years, and median length of time in the United Kingdom before diagnosis was 4 (interquartile range 2–5) years. Of 62 (71%) patients who could be located, 49 participated in extended interviews. Except for homelessness (1 patient), no other social risk factors (e.g., drug and/or alcohol misuse, imprisonment, or mental health problems) were noted. Socializing occurred mainly in places of worship (43/49 patients frequented 9 places of worship) and private homes (10/49 patients frequented 7 residences).

MIRU-VNTR typing was available for all 61 culture-confirmed cases; 46 isolates had 24 loci, and 15 isolates had 15 loci (Table 1). Of the isolates with 24 loci, 27 (59%) clustered in 6 strain types. To identify risk factors for strain-type clustering, we compared demographic characteristics of clustered and nonclustered patients (Table 2). According to bivariable analysis results, a noncongregate location was significantly associated with cluster A only (Table 2).

**Table 1:** *Mycobacterium tuberculosis* strain type clusters involving Eritrea-born patients and associated cluster members born elsewhere, Birmingham, UK, 2004–2013*

Cluster designation (24-loci MIRU-VNTR)†	No. patients in cluster	No. patients born in Eritrea	No. patients born elsewhere (not Eritrea), country‡	No. patients epidemiologically linked to >1 other patient in cluster
A (32433 2242515321 233323462)	12	12	0	12
B (32433 2512511322 132443383)	5	3	2, Yemen	2
C (32435 2332517333 455443382)	3	3	0	2
D (32433 2512511322 131443373)	5	4	1, Kenya	0
E (32432 2311514322 124523342)	3	1	2, Lithuania and Pakistan	2
F (42235 2642515333 342423374)	6	4	2, Pakistan	2
Unique 24-loci strains	NA	18	NA	NA
Only 15 loci available§	NA	15	NA	NA
Culture negative	NA	28	NA	NA
Total	34	88	7	20

*MIRU-VNTR, mycobacterial interspersed repetitive units variable-number tandem-repeat; NA, not applicable. †24-loci MIRU-VNTR was available beginning in 2010. ‡All cases were in recent migrants (arriving in the past 5 y). No epidemiologic links between those from different countries were found except in cluster E. §15-loci MIRU-VNTR typing was available beginning in 2003.

**Table 2:** Demographic and clinical characteristics of 46 Eritrea-born TB patients with 24-loci MIRU-VNTR strain typing available, Birmingham, UK, 2004–2013*

Characteristic	Clustered case, n = 27	Nonclustered case, n = 19	p value†
Median age, y (IQR)	31 (26–37)	29 (26–33)	0.26‡
Male sex, no. (%)	21 (78)	10 (53)	0.11
Regular attendance at noncongregrate location			
Cluster A			<0.01
RV 1	12/12	1/19	
Other venue	0/12	7/19	
Cluster B			0.06
RV 1	1/3	1/19	
Other venue	2/3	7/19	
Cluster C			0.21
RV 1	0/3	1/19	
Other venue	3/3	7/19	
Cluster D			>0.99
RV 1	0/4	1/19	
Other venue	2/4	7/1	
Cluster E			0.45
RV 1	0/1	1/19	
Other venue	1/1	7/1	
Cluster F			>0.99
RV 1	0/4	1/19	
Other venue	2/2	7/1	
Previous BCG vaccination	1 (4)	2 (11)	0.56
History of previous TB	0	0	NA
Pulmonary TB	20 (74)	11 (58)	0.25
Sputum smear positive	6 (30)	4 (36)	>0.99
Isoniazid-monoresistant isolate	3 (11)	1 (5)	0.632

*Data are no. (%) patients unless indicated otherwise. IQR, interquartile range; BCG, **Bacillus Calmette-Guérin;** MIRU-VNTR, mycobacterial interspersed repetitive units variable-number tandem-repeat; NA, not applicable; RV, religious venue; TB, tuberculosis. †Fisher exact test, unless indicated otherwise. ‡Mann-Whitney U test.

The Figure shows the overlap of social networks and MIRU-VNTR strain-type clusters for Eritrea-born TB patients. Cluster A grew from 2 to 12 cases within 8 months. Few cases were linked by routine contact investigation alone because patients named the place of congregation (designated as religious venue [RV] 1 in the Figure) in different ways. The extended interviews defined geographic locations to street level, and it was determined that RV1 was used for multidenominational worship and therefore was recognized by different names. Routine contact investigation also failed to elicit other households where patients socialized (H5–8 in the Figure). As a result of the study, location-based contact tracing at RV1 was undertaken. An additional 68 persons were assessed, of which 19 (28%) had latent TB (16 started treatment and 14 completed treatment) and 1 had active TB. Latent TB was confirmed by a single interferon-γ release assay; therefore, infection resulting from distant exposure could not be ruled out.

**Figure:**
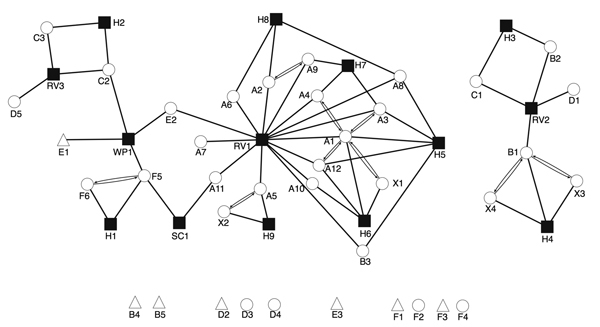
Social network of Eritrea-born patients with TB in relation to 6 distinct 24-loci MIRU-VNTR strain-type clusters and associated cluster members born elsewhere, United Kingdom, January 2009–December 2013. Nonclustered Eritrean patients are included if >1 epidemiologic link to clustered patients is known. Circles denote Eritrea-born patients; triangles denote patients born elsewhere; solid squares denote places of social mixing. For patients, labels denote strain type cluster (A–F; X, no strain typing available) and chronological order of case notification within each strain type cluster. For places, labels denote type (H, private home; RV, religious venue; SC, school; WP, workplace). Patients associated with private homes may or may not usually reside at the address. Double lines with arrows denote connections between TB patients who named each other as contacts during routine contact tracing investigations. Detached symbols at the bottom indicate persons for whom no epidemiologic links to any other case were detected. MIRU-VNTR, mycobacterial interspersed repetitive units variable-number tandem-repeat; TB, tuberculosis.

Combined strain typing and in-depth interviewing also uncovered apparent casual transmission involving cluster E at a workplace (WP1 in the Figure). Six months before diagnosis, patient E2 had worked at WP1 for 2 weeks, during the infectious period of patient E1. As a result, 5 new workplace contacts were investigated; 3 cases were diagnosed (no patients were born in the United Kingdom, and interferon-γ release assay conversion was not documented), and these patients completed treatment for latent TB. A third case (in patient F5) occurred at WP1, 6 months after illness of patient E2, but the isolate from patient F5 was of a different strain type. Given the low probability of transmission, no further location-based contact tracing was undertaken at WP1. Discrepant strain types between patients known to have socialized at RV2, RV3, and school (SC) 1 also suggested that these cases did not involve the same chain of transmission. Thus, no additional contact investigation was undertaken. At RV2, the infectious period for patient B1 preceded the arrival of patient B2 in Birmingham, and local transmission was thought to be unlikely; both patients originated from the same town in Eritrea.

Genetically homogenous strain lineages prevalent in the countries of origin may falsely cluster persons from the migrant population (*10*); in our study, no patients within cluster D could be linked despite extensive epidemiologic investigation. However, microepidemics within such clusters do occur. 

## Conclusions

We have demonstrated the value of identifying places of social mixing during contact investigation for recognizing such microepidemics early. This approach has been found useful in other settings (*11*,*12*). A recent evaluation of the TB strain typing service in the United Kingdom found that strain typing did not significantly affect time to diagnosis or the median number of secondarily infected persons found per index case (*13*). However, strain typing can highlight gaps in current contact investigation procedures in specific patient populations and can help focus resources on scenarios in which recent transmission is more likely. Whole-genome sequencing may offer the ability to identify more recent strain evolution and transmission (*14*).

TB services should be vigilant for emergence of TB microepidemics in new communities. Conventional epidemiologic methods should be improved to complement molecular epidemiologic methods and increase their effect on TB control.
